# Theory of Mind and Psychosocial Characteristics in Older Men

**DOI:** 10.1037/pag0000324

**Published:** 2018-12-20

**Authors:** Marcin A. Radecki, Simon R. Cox, Sarah E. MacPherson

**Affiliations:** 1 Human Cognitive Neuroscience and Department of Psychology, University of Edinburgh; 2 Centre for Cognitive Ageing and Cognitive Epidemiology and Department of Psychology, University of Edinburgh; 3 Human Cognitive Neuroscience, Centre for Cognitive Ageing and Cognitive Epidemiology and Department of Psychology, University of Edinburgh

**Keywords:** theory of mind, Faux Pas test, psychosocial characteristics, individual differences, older age

## Abstract

The extent to which early-life cognitive ability shapes individuals’ social functioning throughout life, in the context of later-life factors, is unknown. We investigated performance on the Faux Pas test (FP) in relation to psychosocial characteristics and childhood intelligence scores in 90 healthy older men. FP performance was associated with close social network size but not social contact, social support, or loneliness when accounting for both childhood and later-life intelligence, affect, personality, and sociodemography. We add to a growing literature on associations between theory of mind and intelligence, affect, and personality.

Socioemotional selectivity theory (Carstensen, 2006) posits that older individuals strategically prune their social networks in favor of social environments that are more emotionally satisfying (English & Carstensen, 2014). The positive emotions experienced by older adults during social interactions are thought to improve their emotional well-being (Scheibe & Carstensen, 2010) and social functioning (Charles & Carstensen, 2010; Luong, Charles, & Fingerman, 2011).

The formation and maintenance of successful social relationships in older adults may also rely on theory of mind (ToM). ToM is the ability to ascribe mental states to oneself and others to explain and predict behavior (Premack & Woodruff, 1978). Deficits in performance-based ToM correspond to impairments in real-world social functioning among clinical groups (Bishop-Fitzpatrick, Mazefsky, Eack, & Minshew, 2017) and typically developing children (Caputi, Lecce, Pagnin, & Banerjee, 2012). However, while ToM declines in older age (Henry, Phillips, Ruffman, & Bailey, 2013), the role that ToM plays in older adults’ social lives remains poorly understood.

In one of the few studies exploring the relationship between ToM and psychosocial functioning in later adulthood, Bailey, Henry, and Von Hippel (2008) demonstrated that reduced mental state understanding was associated with decreased social participation in older adults. Yeh (2013) later showed that ToM performance was related to self-reported social skills in older age. Also, Lecce et al. (2017) found links between older adults’ ToM performance and self-perceived friendship indicators. Nonetheless, not only does the robustness of these associations require further investigation, but also whether they are potentially modulated by individual differences in nonsocial psychological domains.

The correspondence between ToM and psychosocial characteristics might be partly affected by cognitive variation. The two-systems account of ToM proposes that mental state understanding relies on both the readily triggered implicit processes of sensory perception (e.g., face processing) and the consciously guided explicit processes of higher cognitive skills, such as language and reasoning (Apperly & Butterfill, 2009; Frith & Frith, 2008). Indeed, ToM has been related to verbal ability and abstract reasoning in young adults (Ahmed & Stephen Miller, 2011), and abstract reasoning in older adults (Cox et al., 2014). Meinhardt-Injac, Daum, Meinhardt, and Persike (2018) found that a verbal latent factor, but not an abstract reasoning one, predicted ToM abilities in young adults. To our knowledge, however, the independent influences of language and abstract reasoning on associations between ToM and psychosocial characteristics have not been examined in older age.

Furthermore, while certain cognitive functions (including fluid intelligence) deteriorate, on average, with age (Singh-Manoux et al., 2012), individual differences in intelligence remain relatively stable from childhood to later adulthood (Deary, 2014). While such measures of general cognitive ability are related to ToM in children (Ibanez et al., 2013) and older adults (Cox et al., 2014; this sample), the lifelong stability of intelligence poses questions about how much later-life ToM and social support might simply reflect a lifelong association between early-life intelligence and social abilities. However, this possibility, in the context of other important later-life factors, has yet to be investigated.

Links between ToM and psychosocial factors may also be influenced by affect and personality. Major depressive disorder (MDD; Bora & Berk, 2016) and social anxiety disorder (Washburn, Wilson, Roes, Rnic, & Harkness, 2016) have been coupled with ToM impairments in young and/or older adulthood. Regarding personality, Agreeableness, for example, has been shown to correlate with ToM performance in young adults (alongside Neuroticism; Nettle & Liddle, 2008) and moderate day-to-day interpersonal conflicts in adolescents (Jensen-Campbell & Graziano, 2001). Yet, the effects of affect and personality on links between ToM and psychosocial functioning remain seemingly unexplored in older age.

The Faux Pas test (FP; Stone, Baron-Cohen, & Knight, 1998) is a widely used task that is administered to assess age-related decline in ToM (Henry et al., 2013). Participants are presented with 20 written stories, 10 of which contain a faux pas. Participants should identify whether the protagonists’ spoken words may have unintentionally upset other characters in the stories; this requires an understanding of others’ cognitive and affective states.

The present study aimed to characterize relationships between ToM, as measured by the FP test, and psychosocial characteristics in older age, where impaired mental state understanding putatively entails decreased social participation. The second goal was to determine whether early-life cognitive ability influences the relationship between ToM and psychosocial characteristics in later-life or whether there are additional influences from later-life nonsocial factors, such as crystallized (*g*_c_) and fluid (*g*_f_) intelligence, depressive and anxious symptoms, and personality traits, but also sociodemography.

## Method

### Participants

Ninety males aged 73.2 to 75.0 years from the Lothian Birth Cohort 1936 (LBC1936) participated. Cohort members were born in 1936 and tested at age ∼11 on the Moray House Test (MHT) for the Scottish Mental Survey of 1947 (Deary, Whalley, & Starr, 2009; Scottish Council for Research in Education, 1949). Raw MHT scores (maximum = 76) were converted into intelligence scores (*M* = 100, *SD* = 15) and corrected for age (in days) at testing. Participants were recruited from Edinburgh and surrounding areas for follow-up testing at age ∼70 years (Wave 1; Deary et al., 2007), and returned 3 years later for further testing (Wave 2; Deary, Gow, Pattie, & Starr, 2012).

The current sample was invited to participate in a cortisol study during which the Faux Pas test was administered (Cox, Bastin, et al., 2015; Cox et al., 2014, 2017; Cox, MacPherson, et al., 2015). The inclusion criteria included: male (due to sex differences in glucocorticoid secretion patterns), ≥ 24 on the Mini-Mental State Examination (Folstein, Folstein, & McHugh, 1975); < 11 on the Depression subscale of the Hospital Anxiety and Depression Scale (HADS; Zigmond & Snaith, 1983), a structural brain MRI scan within 1.5 years, and no current antidepressant or glucocorticoid medication use. Ethical permission was obtained from the Multi-Centre Research Ethics Committee for Scotland (MREC/01/0/56), the Lothian Research Ethics Committee (LREC/2003/2/29), and the Scotland A Research Ethics Committee (07/MRE00/58). All volunteers gave written informed consent.

At Wave 2, participants indicated their relationship status, dichotomously coded as single/divorced/widowed (0) versus married/cohabiting/other (1), and living arrangement, dichotomously coded as living alone (0) versus living with others (1). An index of local deprivation (Scottish Index of Multiple Deprivation; SIMD; Scottish Executive, 2004) was derived for each participant at Wave 1, assessing employment, income, health, education, access, crime, and housing. SIMD (henceforth, reported as local deprivation) was measured on a ratio scale, with a score of 1 indicating the most and 6,505 the least deprived data zone.

### Measures

#### The FP test

Wave 2 participants undertook the FP test individually. Ten of the 20 stories contained a verbal faux pas. Participants read each story at their own pace and indicated when they were finished. They were then read a series of eight questions while the story remained in front of them. The first assessed whether participants recognized that a faux pas had been committed. The next four assessed cognitive ToM: who committed the faux pas, the inappropriateness of what was said, the speaker’s motivations, and a story character’s beliefs. The final question assessed empathy/affective ToM: how the characters felt. Finally, two control questions assessed whether participants understood the story. The main outcome variable was the total number of correct responses for the 10 faux pas stories, excluding responses to the empathy/affective ToM and control questions. Each correct response was awarded 1 point (maximum = 50). Scoring was according to the Stone et al. (1998) guidelines. The FP test is considered a valid measure of ToM (Baron-Cohen, O’Riordan, Stone, Jones, & Plaisted, 1999), and has good internal consistency (Cronbach’s alpha = .91) and test–retest reliability (Cronbach’s alpha = .89; Yeh, Hua, & Liu, 2009).

#### Social contact

Wave 1 participants completed eight yes/no items relating to social life (Gow, Corley, Starr, & Deary, 2013). The four questions regarding social contact (Cronbach’s alpha = .67)—such as: “In the last 2 weeks, excluding people you live with, have you seen a friend to have a chat to?”—were summed (for all questions, see Table 1 in the online supplemental materials). Higher scores indicated greater social contact.

#### Social support

Wave 1 participants completed a 12-item questionnaire adapted from the Social Support Questionnaire (Short Form; SSQ; Sarason, Sarason, Shearin, & Pierce, 1987). It comprised six questions regarding the level of social support received, measured on a 5-point scale from *None of the time* to *All of the time*, such as: “How often could you count on people to console you when you were very upset?” (for all questions, see Table 2 in the online supplemental materials). Higher scores indicated greater social support.

#### Loneliness

Wave 2 participants were asked from the European Social Survey (2006): “How often have you felt lonely during the past week?” The item was measured on a 4-point scale ranging from *None or almost none of the time* to *All or almost all of the time*. Since 74 out of 90 participants (82.2%) reported feeling lonely none or almost none of the time, the data were collapsed into a dichotomous variable coded as not lonely (0) versus lonely (1).

#### Close social network size

Wave 1 participants were asked to estimate the size of their close social networks in response to: “About how many ‘close’ friends and ‘close’ relatives do you have (‘close’ meaning people that you feel at ease with, could talk to about what was on your mind, and could call on for help)?”

#### Crystallized intelligence (*g*_c_)

To measure *g*_c_, the Wechsler Test of Adult Reading (Wechsler, 2001) was administered at Wave 2. It requires the pronunciation of 50 irregularly spelled words; 1 point for each correct pronunciation was awarded, and a score out of 50 was used as the outcome variable.

#### Fluid intelligence (*g*_f_)

At Wave 2, *g*_f_ was assessed using five subtests from the Wechsler Adult Intelligence Scale-III UK (WAIS-III UK; Wechsler, 1997a): Block Design, Letter-Number Sequencing, Matrix Reasoning, Digit Symbol, and Symbol Search; and one subtest from the Wechsler Memory Scale-III UK (WMS-III UK; Wechsler, 1997b): Backward Digit Span. In all subtests, higher scores indicated higher *g*_f_.

#### Depression and anxiety

The HADS (Zigmond & Snaith, 1983) was completed at Wave 2, with seven items examining symptoms of depression and seven of anxiety. Both subscales had a maximum score of 21; higher values represented more depressive and anxious symptoms.

#### Personality

The International Personality Item Pool (IPIP; Goldberg, 1999) was administered at Wave 2 to assess Big-Five personality traits (Costa & McRae, 1992): Extraversion, Agreeableness, Conscientiousness, Emotional Stability (reverse of Neuroticism), and Intellect (henceforth, reported as Openness). Higher scores indicated greater personality trait presence, and each domain allowed for a maximum score of 40.

See Table 3 in the online supplemental materials for the assessment wave for each study variable.

### Statistical Analyses

Statistical analyses were performed in R Version 3.2.2 (“Fire Safety”; R Core Team, 2016). Social support was derived by entering the six SSQ questions (Cronbach’s alpha = .94) into a principal component analysis (PCA) using the “principal” function from the “psych” package, using data from all LBC1936 Wave 1 participants (*N* = 1,091), from which the scores for the FP subsample (*N* = 90) were extracted. The first unrotated principal component accounted for 76% of the variance (all loadings ≥ .84; see Table 2 in the online supplemental materials).

To derive a general measure of *g*_f_, a PCA was conducted using five WAIS-III UK (Block Design, Letter-Number Sequencing, Matrix Reasoning, Digit-Symbol, and Symbol Search) and one WMS-III UK subtests (Backward Digit Span), using all available data from the LBC1936 at Wave 2 and then extracting the scores for the FP subsample. The first unrotated principal component accounted for 51% of the variance (Cronbach’s alpha = .73; all loadings > .65; see Table 4 in the online supplemental materials).

We used Welch’s *t* tests (for ratio variables) and Pearson’s chi-squared tests (for categorical and ordinal variables) to examine whether those who performed the FP test (*N* = 90) differed significantly from those who did not (*N* = 1,001) on psychosocial, cognitive, affective, personality, and sociodemographic data. Next, we examined bivariate associations between all study variables (Pearson’s *r*).

We then used multiple linear regression—simultaneously modeling intelligence, affect, personality, and sociodemography—to ascertain the unique contributions each variable made to FP performance. Living arrangement was dropped from the model, given its high variance inflation alongside relationship status. Then, we regressed MHT scores onto *g*_c_ and *g*_f_, and ran the model again with these residuals, thereby accounting for childhood intelligence. Finally, associations between FP performance and our psychosocial outcomes, treated as dependent variables, were explored using hierarchical regression. Separate models were fitted for each psychosocial variable to examine the variance accounted for by FP performance alongside other potentially confounding measures. With each model iteration, predictors were added incrementally: (a) FP performance, (b) age (in days), (c) intelligence, (d) symptoms of depression and anxiety, (e) five personality traits, and (f) relationship status and local deprivation (again, living arrangement was not included). We used multiple linear regression except for loneliness, where we used binomial logistic regression. The *p* values were corrected for multiple comparisons using false discovery rate (FDR; Benjamini & Hochberg, 1995) with “p.adjust” from the “stats” package. Variance inflation was ascertained for each model fit using “vif” from the “car” package. Finally, we regressed MHT scores onto *g*_c_ and *g*_f_, and ran the models again using these residuals to take account of childhood intelligence.

## Results

Descriptive statistics and tests of differences between the FP subsample and the LBC1936 (excluding the FP subsample) are reported in Table 5 in the online supplemental materials.

Bivariate associations between the study variables within the FP subsample are shown in Table 1. They revealed that better FP performance correlated with higher MHT scores, *r* = .591, *p* < .001, higher *g*_c_, *r* = .588, *p* < .001, higher *g*_f_, *r* = .482, *p* < .001, higher Openness, *r* = .311, *p* = .003 and Emotional Stability, *r* = .222, *p* = .036, and fewer depressive symptoms, *r* = −.278, *p* = .008. Better FP performance also correlated with smaller close social network size, *r* = −.397, *p* = < .001, but not with any other psychosocial characteristic (*r*s ≤ .199, *p*s ≥ .060).[Table-anchor tbl1]

Using multiple regression, only *g*_c_ (β = .367, *p* < .001), *g*_f_ (β = .216, *p* = .026), and depressive symptoms (β = −.227, *p* = .025) made significant unique contributions to FP performance, with the overall model accounting for 39% of the total variance. The only variable to survive FDR correction (*q* < .007) was *g* (see Table 6 in the online supplemental materials). When considering childhood intelligence, *g*_c_ and *g*_f_ no longer significantly contributed to FP performance (*p* ≥ .09). Only depressive symptoms (β = −.277, *p* = .030) were a significant predictor, but this did not survive FDR correction (see Table 7 in the online supplemental materials).

Finally, we determined whether FP performance predicted psychosocial characteristics (Table 2). Higher FP performance was significantly associated with smaller close social network size in all model iterations, and this relationship survived correction for multiple testing (β range = −.501 to −.435, *p* value range ≤ .001 to .010). Better FP performance was also significantly related to lower loneliness in models correcting for age and intelligence (odds ratio [*OR*] = 0.382, 95% CI [0.167, 0.777], *p* = .012), and this relationship was robust to multiple testing correction. There were no associations between FP performance and social contact or social support, and these remained nonsignificant with each stepwise model iteration (β ≤ .176, *p*s ≥ .247). When including childhood intelligence, higher FP performance remained significantly associated with smaller close social network size in all model iterations (β range = −.574 to −.475, *p* value range ≤ .001 to .002). However, the models predicting loneliness were no longer significant (*OR* ≤ .742, *p*s ≥ .072; see Table 8 in the online supplemental materials).[Table-anchor tbl2]

## Discussion

This study focused on whether ToM, measured using the FP test, was related to psychosocial characteristics in 90 healthy older men. It further explored whether early-life cognitive ability influences the relationship between ToM and psychosocial characteristics in older age or whether there are additional influences from later-life cognitive, affective, personality, and sociodemographic factors. In bivariate analyses, better FP performance was associated with higher scores on measures of *g*_c_, *g*_f_, and the personality traits of Openness and Emotional Stability, as well as fewer depressive symptoms. When all potential predictors of FP performance were considered in a simultaneous regression analysis, *g*_c_, *g*_f_, and depressive symptoms made significant unique contributions—but only *g*_c_ survived correction for multiple testing. However, neither *g*_c_ nor *g*_f_ predicted FP performance when childhood intelligence was considered, suggesting that lifelong, stable differences in cognitive ability underlie the relationships between ToM and later-life intelligence.

We also found that individuals more able to detect social slips reported having smaller close social networks and being less lonely. Importantly, those children with lower intelligence grew up to have lower FP scores and greater loneliness; however, it appears that childhood intelligence did not contribute to the relationship between higher FP scores and smaller close social network size.

The association between FP performance and close social network size suggests that individuals with greater social-cognitive aptitude build smaller close social circles, or that poorer social reasoning relates to maintaining a higher number of close others (at an age when social relationships generally reduce; Lang & Carstensen, 1994). Whereas those individuals with lower childhood intelligence were at risk of greater loneliness and poorer ToM performance, childhood intelligence did not account for links between FP scores and close network size. Early-life intelligence does not appear to have a significant impact on the ability to successfully prune one’s network to maximize emotional satisfaction (Carstensen, 2006).

Another finding was the significant link between FP performance and *g*_c_ and *g*_f_. In simple bivariate analyses, both were associated with FP performance with comparable magnitudes; the FP-*g*_f_ association supports prior findings (Cox et al., 2014; Ibanez et al., 2013), but the FP-*g*_c_ relationship had not been examined. Here, when *g*_f_ was modeled simultaneously alongside other covariates, *g*_c_ remained a significant FP predictor, corroborating prior evidence that verbal ability uniquely moderates various indices of ToM (Meinhardt-Injac et al., 2018). It is also relevant to reports that reading literary fiction may enhance social reasoning in adults (Kidd & Castano, 2013). Our study’s advantage was that it was able to demonstrate that the relationship between intelligence and ToM begins in childhood and persists throughout life.

FP performance was also correlated with self-reported depressive symptoms, as measured by the HADS. This is in line with research indicating that current depressive symptoms or diagnosed MDD debilitate ToM regardless of age (Bora & Berk, 2016). Moreover, depressive symptoms were a nominally significant predictor of FP performance beyond the variance this task shares with intelligence itself.

Finally, Openness and Emotional Stability, measured using the IPIP, were the Big-Five personality dimensions significantly correlated with FP performance. A similar relationship between Openness and ToM has been reported in younger age (Mar, Oatley, & Peterson, 2009; but see Nettle & Liddle, 2008). Higher Openness might relate to greater cognitive ability (Soubelet & Salthouse, 2011), which is supported by our finding that the association between Openness and FP performance became nonsignificant when accounting for intelligence. Similarly, Emotional Stability was no longer significantly linked with FP performance when entered into this regression model, which also included depressive and anxious symptomatology. Future research should further address how personality relates to ToM using various tasks while accounting for intelligence and affect.

Our study has several limitations. Importantly, we used only one ToM measure. Additionally, it would have been optimal to determine the influence of psychosocial characteristics on FP performance compared with control stories. However, participants performed almost entirely at ceiling on these, which is common in studies using the FP test (MacPherson, Phillips, & Della Sala, 2002; Stone et al., 1998). This indicates that individual FP variability is unlikely to be attributable to participants’ lack of story comprehension. Our close social network size data were cross-sectional and so we cannot directly address the phenomenon of network size pruning. Moreover, two measures were based on single items (loneliness and close social network size) and some were binary (loneliness, relationship status, and living arrangement), reducing fidelity to capture individual variation. Also, loneliness was unbalanced and underpowered, limiting the reliability to estimate its predictors. Furthermore, inclusion of solely male participants within a narrow old-age range, and the restricted nature of this cohort study (Johnson, Brett, Calvin, & Deary, 2016), limit the generalizability of our results to females, other ages, and, perhaps, less healthy participants of the same age. Finally, while our study was sufficiently powered to reliably detect large- and medium-sized effects, our sample size precluded the reliable estimation of small effects and may have resulted in overfitting and inflation of effect sizes (Ioannidis, 2008).

Notwithstanding these limitations, we provide evidence that ToM, or at least the FP test, may be selectively sensitive to psychosocial characteristics in older age; those more aware of social slips were more likely to report fewer close others, and to report being less lonely, but were not more or less socially isolated with respect to social contact or support. Future work might consider whether ToM contributes to the investment in maintaining closer, more positive relationships as individuals age. However, our work is preliminary and should be interpreted with caution until replicated in wider and more varied samples.

## Supplementary Material

10.1037/pag0000324.supp

## Figures and Tables

**Table 1 tbl1:** Correlation Matrix of the Study Variables

Study variables	1	2	3	4	5	6	7	8	9	10	11	12	13	14	15	16	17	18
1. Faux Pas performance		.591	−.127	.024	−.397	−.199	.588	.482	−.157	−.278	.167	.065	.085	.222	.311	−.020	−.009	.190
2. MHT	<.001		−.127	−.085	−.225	.114	.767	.695	−.204	−.111	.088	−.001	−.058	.132	.439	−.031	.027	.405
3. Social contact	.259	.278		.168	.228	.086	−.074	.066	.077	−.002	.054	.098	.095	−.049	.006	.278	.103	.033
4. Social support	.830	.466	.133		.188	−.254	−.009	.112	−.171	−.420	.168	.223	.097	.133	−.059	.225	.259	−.069
5. Close social net. size	<.001	.054	.042	.096		−.176	−.209	−.072	−.003	.017	.041	−.025	.141	−.026	−.042	.201	.146	−.152
6. Loneliness	.060	.310	.448	.022	.119		−.008	.076	.362	.377	−.391	−.065	−.335	−.425	.030	−.201	−.140	−.036
7. *g*_c_	<.001	<.001	.513	.936	.064	.943		<.001	−.153	−.150	.111	.048	.002	.116	.430	−.004	.036	.387
8. *g*_f_	<.001	<.001	.557	.318	.526	.475	.608		−.128	−.091	.039	−.003	−.004	.083	.285	.122	.087	.356
9. Anxiety	.139	.066	.494	.128	.981	<.001	.153	.228		.515	−.347	−.221	−.258	−.742	−.060	−.108	−.099	−.039
10. Depression	.008	.323	.989	<.001	.880	<.001	.160	.396	<.001		−.333	−.189	−.238	−.484	−.091	−.272	−.277	−.032
11. Extraversion	.116	.433	.633	.133	.717	<.001	.300	.717	.001	.001		.330	.277	.469	.360	−.019	−.009	−.029
12. Agreeableness	.543	.949	.385	.046	.826	.541	.658	.977	.036	.074	.001		.208	.319	.223	−.091	−.032	−.102
13. Conscientiousness	.425	.603	.397	.389	.211	.001	.983	.967	.014	.024	.008	.049		.327	.210	−.024	−.046	.084
14. Emotional Stability	.036	.238	.662	.235	.819	<.001	.279	.435	<.001	<.001	<.001	.002	.002		.153	.079	.105	−.009
15. Openness	.003	<.001	.958	.603	.708	.780	<.001	.006	.571	.394	<.001	.035	.047	.151		−.205	−.104	.091
16. Relationship status	.853	.782	.012	.043	.074	.057	.969	.251	.312	.009	.862	.393	.821	.462	.053		.870	.047
17. Living arrangement	.931	.807	.359	.020	.196	.189	.739	.412	.353	.008	.931	.763	.664	.326	.330	<.001		.029
18. Local deprivation	.075	<.001	.772	.541	.183	.735	<.001	.001	.715	.767	.785	.342	.436	.934	.394	.661	.784	
*Note*. Pearson’s *r*s (upper diagonal) and *p*s (lower diagonal) are reported for associations within the Faux Pas subsample (*N* = 90). MHT = Moray House Test; Close social net. size = close social network size; g_c_ = crystallized intelligence; *g*_f_ = fluid intelligence.																		

**Table 2 tbl2:** Regression Results for Faux Pas Performance as a Predictor of Psychosocial Characteristics

Model	β	*SE*	*t*	*p*
Social contact				
FP	−.122	.107	−1.14	.259
FP + Age	−.123	.107	−1.15	.256
FP + Age + Intelligence	−.143	.141	−1.01	.314
FP + Age + Intelligence + Affect	−.176	.151	−1.17	.247
FP + Age + Intelligence + Affect + Personality	−.161	.156	−1.03	.305
FP + Age + Intelligence + Affect + Personality + Sociodemography	−.130	.154	−.85	.400
Social support				
FP	.025	.115	.22	.830
FP + Age	.025	.116	.21	.832
FP + Age + Intelligence	.047	.153	.31	.758
FP + Age + Intelligence + Affect	−.152	.147	−1.03	.306
FP + Age + Intelligence + Affect + Personality	−.137	.151	−.91	.366
FP + Age + Intelligence + Affect + Personality + Sociodemography	−.139	.153	−.91	.368
Close social network size				
FP	−.499	.131	−3.82	**<.001**
FP + Age	−.501	.124	−4.03	**<.001**
FP + Age + Intelligence	−.440	.159	−2.76	**.007**
FP + Age + Intelligence + Affect	−.501	.169	−2.97	**.004**
FP + Age + Intelligence + Affect + Personality	−.476	.175	−2.73	**.008**
FP + Age + Intelligence + Affect + Personality + Sociodemography	−.435	.164	−2.65	**.010**
	*OR*	2.5%	97.5%	*p*
Loneliness				
FP	.646	.397	1.053	.072
FP + Age	.647	.397	1.054	.072
FP + Age + Intelligence	.382	.167	.777	**.012**^a^
FP + Age + Intelligence + Affect	.386	.149	.882	.032
FP + Age + Intelligence + Affect + Personality	.357	.112	.927	.048
FP + Age + Intelligence + Affect + Personality + Sociodemography	.329	.098	.884	.040
*Note*. Bold typeface denotes significant *p* values following false discovery rate correction across all results reported in the table. All variance inflation factors ≤ 2.830. β = standardized coefficients reported except for loneliness (odds ratios [*OR*s)]; FP = Faux Pas performance; Intelligence = fluid intelligence and crystallized intelligence; Affect = symptoms of depression and anxiety; Personality = Extraversion, Agreeableness, Conscientiousness, Emotional Stability, and Openness; Sociodemography = relationship status and local deprivation.				
^a^ No longer significant (*p* = .076) when controlling for childhood intelligence.				
