# Supplementary material for: Theory of Mind and Psychosocial Characteristics in Older Men
Source: Psychol Aging. 2018 Dec 20;34(1):145–51. doi: 10.1037/pag0000324 (PMC6366441; doi:10.1037/pag0000324)
Supplement: Supplementary file 1 [file PAG-2018-0072Suppl.docx]

**Supplemental Materials**

*Supplemental Table 1.* Social contact items

| *Items* |
| --- |
| In the last 2 weeks, excluding people you live with, have you seen a friend to have a chat to?  In the last 2 weeks, excluding people you live with, have you seen someone in your family to chat to?  In the last 2 weeks, excluding people you live with, have you had contact by telephone or letter with a friend?  In the last 2 weeks, excluding people you live with, have you had contact by telephone or letter with your family? |

*Note.* The above items (Cronbach’s α = .67) were summed.

*Supplemental Table 2.* Component loadings of the social support items

| \| *Items* \| *Loadings* \| \| --- \| --- \| \| How often were there people you could really count on to be dependable when you needed help? \| .84 \| \| How often were there people you could really count on to help you feel more relaxed when you were under pressure or tense? \| .87 \| \| How often were there people who accepted you totally, including both your worst and your best points? \| .84 \| \| How often could you really count on people to care about you, regardless of what was happening to you? \| .88 \| \| How often could you really count on people to help you feel better when you were feeling generally down-in-the-dumps? \| .91 \| \| How often could you count on people to console you when you were very upset? \| .90 \| |
| --- | --- | --- | --- | --- | --- | --- | --- | --- | --- | --- | --- | --- | --- | --- |

*Note.* Component loadings are given for the first unrotated principal component from a Principal Components Analysis (PCA) of the above items from the Social Support Questionnaire (Short Form; SSQ) (Cronbach’s α = .94), explaining 76% of the total variance.

*Supplemental Table 3*. Assessment wave for each study variable

| *Variables* | *Waves* |
| --- | --- |
| Faux Pas test | 2 |
| Social contact | 1 |
| Social support | 1 |
| Loneliness | 1 |
| Close social network size | 1 |
| *g*_c_ | 2 |
| *g*_f_ | 2 |
| Anxiety and depression | 2 |
| Personality  Relationship status  Living arrangement  Local deprivation | 2  2  2  1 |

*Note.* Wave 1 = approximately aged 70 years; Wave 2 = approximately aged 73 years. g_c_ = crystallized intelligence; *g*_f_ = fluid intelligence.

*Supplemental Table 4*. Component loadings of the *g*_f_ items

| \| *Items* \| *Loadings* \| \| --- \| --- \| \| Backward Digit Span \| .65 \| \| Block Design \| .72 \| \| Letter-Number Sequencing \| .71 \| \| Matrix Reasoning \| .70 \| \| Digit Symbol Substitution \| .75 \| \| Symbol Search \| .76 \| |
| --- | --- | --- | --- | --- | --- | --- | --- | --- | --- | --- | --- | --- | --- | --- |

*Note.* Component loadings are given for the first unrotated principal component from a PCA of the above subtests from the Wechsler Adult Intelligence Scale-III (UK) and Wechsler Memory Scale-III (UK) (Cronbach’s α = .73), explaining 51% of the total variance.

*Supplemental Table 5.* Characteristics of the Lothian Birth Cohort 1936 (LBC1936) and the Faux Pas subsample

| *Sample* | *LBC1936 (N = 1001)* | | | *Faux Pas (N = 90)* | | |  |
| --- | --- | --- | --- | --- | --- | --- | --- |
|  | *M* | *SD* | *Range* | *M* | *SD* | *Range* | *p* |
| Faux Pas test | − | − | − | 39.3 | 7.7 | 12-49 | − |
| Age (years) | 72.4 | 0.7 | 70.9-74.2 | 73.1 | 0.4 | 72.2-74.1 | < .001 |
| MHT | 100.0 | 14.9 | 38.5-129.9 | 99.9 | 15.8 | 60.9-125.9 | .957 |
| MMSE  Social contact  Social support  Close social network size  Loneliness  *g*_c_  *g*_f_  Anxiety  Depression  Extraversion  Agreeableness  Conscientiousness  Emotional stability  Openness  Relationship status | 28.8  3.7  0  106  −  41.0  0.0  4.6  2.6  21.7  31.0  27.8  24.9  23.8  − | 1.4  0.6  1.0  7.9  −  6.9  1.0  3.2  2.2  7.2  5.6  6.1  7.6  6.0  − | 20-30  0-4  −4.5-0.9  0-70  636:129  5-50  −3.5-2.9  0-19  0-13  0-40  10-40  5-40  2-40  5-40  232:544^c^ | 28.6  3.8  −0.2  12.3  −  41.1  0.0  4.0  2.7  20.7  29.1  27.0  25.9  22.9  − | 1.5  0.6  1.0  9.8  −  7.9  1.1  2.7  2.3  7.5  5.1  6.0  8.2  5.6  − | 24-30  2-4  −3.6-0.9  1-50  74:16  22-50  −2.5-3.2  0-10  0-10  2-34  15-40  6-38  7-40  8-37  14:76^c^ | .232  .911^a^  .099  .144  .944^a^  .942  .786  .047  .608  .214  .002  .251  .282  .135  .006^a^ |
| Living arrangement | − | − | 212:563^d^ | − | − | 13:77^d^ | .012^a^ |
| Local deprivation | 4556.6 | 1925.4 | 4-6505 | 4655.9 | 1699.1 | 788-6504 | .603 |

*Note*. *M* = mean; *SD* = standard deviation; ^a^Pearson’s chi-squared test *p*-values of group differences reported (otherwise, Welch’s t-test *p*-values reported); ^b^ notlonely:lonely, ^c^ single/divorced/widowed:married/cohabiting/other; ^d^ living alone:living with others; MHT = Moray House Test; MMSE = Mini Mental State Examination; g_c_ = crystallized intelligence; *g*_f_ = fluid intelligence.

| *Supplemental Table 6.* Multiple linear regression results for predictors of Faux Pas performance | | | | |
| --- | --- | --- | --- | --- |
|  | ***β*** | ***SE*** | ***t*** | ***p*** |
| *g*_c_  g_f_  Anxiety  Depression  Extraversion  Agreeableness  Conscientiousness  Emotional stability  Openness  Relationship status  Local deprivation | .367  .216  .223  −.227  .010  −.058  .007  .184  .012  −.263  −.061 | .104  .095  .152  .100  .101  .102  .095  .127  .112  .250  .105 | 3.55  2.27  1.47  −2.28  0.10  −0.57  0.07  1.45  0.11  −1.05  −0.58 | < .001  .026  .146  .025  .922  .573  .941  .150  .914  .296  .563 |

*Note.* *β *= standardized coefficients. All variance inflation factors ≤ 2.64, except for relationship status (4.67) and living arrangement (4.41), not reported in the table. g_c_ = crystalized intelligence; *g*_f_ = fluid intelligence.

| *Supplemental Table 7.* Multiple linear regression results for predictors of Faux Pas performance when g_c_ and *g*_f_ were regressed for childhood intelligence | | | | |
| --- | --- | --- | --- | --- |
|  | ***β*** | ***SE*** | ***t*** | ***p*** |
| *g*_c_ | .167 | .110 | 1.52 | .133 |
| *g*_f_  Anxiety | .180  .171 | .103  .189 | 1.75  0.91 | .086  .369 |
| Depression | −.277 | .125 | −2.21 | .030 |
| Extraversion | −.034 | .125 | −0.27 | .788 |
| Agreeableness | −.049 | .122 | −0.40 | .689 |
| Conscientiousness | −.062 | .112 | −0.55 | .584 |
| Emotional stability | .194 | .159 | 1.22 | .227 |
| Openness | .208 | .124 | 1.67 | .099 |
| Relationship status | −.195 | .315 | −0.62 | .538 |
| Local deprivation | .148 | .127 | 1.17 | .246 |

*Note.* *β* = standardised coefficients. All variance inflation factors ≤ 2.79. g_c_ = crystalized intelligence; *g*_f_ = fluid intelligence. The overall model accounted for 17% of the total variance.

| *Supplemental Table 8.* Regression results for Faux Pas performance as a predictor of psychosocial characteristics when the intelligence predictors were regressed for childhood intelligence | | | | |
| --- | --- | --- | --- | --- |
|  | ***β*** | ***SE*** | ***t*** | ***p*** |
| *Social contact*  FP  FP + age  FP + age + intelligence  FP + age + intelligence + affect  FP + age + intelligence + affect + personality  FP + age + intelligence + affect + personality + socio-demography  *Social support*  FP  FP + age  FP + age + intelligence  FP + age + intelligence + affect  FP + age + intelligence + affect + personality  FP + age + intelligence + affect + personality + socio-demography  *Close social network size*  FP  FP + age  FP + age + intelligence  FP + age + intelligence + affect  FP + age + intelligence + affect + personality  FP + age + intelligence + affect + personality + socio-demography  *Loneliness* | −.122  −.123  −.263  −.292  −.253  −.242  .025  .025  .021  −.163  −.128  −.119  −.499  −.501  −.511  −.574  −.529  −.475  ***OR*** | .107  .107  .116  .124  .129  .127  .115  .116  .139  .132  .138  .141  .131  .124  .144  .153  .159  .150  **2.5%** | −1.14  −1.15  −2.26  −2.36  −1.95  −1.91  0.22  0.21  0.15  −1.24  −0.93  −0.84  −3.82  −4.03  −3.56  −3.75  −3.32  −3.17  **97.5%** | .259  .256  .027  .021  .055  .061  .830  .832  .882  .221  .356  .402  **< .001**  **< .001**  **< .001**  **< .001**  **.002**  **.002**  ***p*** |
| FP  FP + age  FP + age + intelligence  FP + age + intelligence + affect  FP + age + intelligence + affect + personality  FP + age + intelligence + affect + personality + socio-demography | 0.646  0.647  0.608  0.742  0.500  0.539 | 0.397  0.397  0.342  0.403  0.214  0.223 | 1.053  1.054  1.056  1.381  1.080  1.214 | .072  .072  .076  .328  .082  .138 |

*Note.* *β* = standardized coefficients except for loneliness (odds ratios); FP = Faux Pas performance; intelligence = Moray House Test scores regressed onto *g*_c_ and *g*_f_; affect = symptoms of depression and anxiety; personality = extraversion, agreeableness, conscientiousness, emotional stability, and openness; socio-demography = relationship status and local deprivation. Bold typeface denotes significant *p*-values following False Discovery Rate correction across all results reported in the table. All variance inflation factors ≤ 2.810.
